# From transcriptomic profiling to precision oncology: a bibliometric analysis of RNA sequencing in acute myeloid leukemia

**DOI:** 10.3389/fmed.2026.1877223

**Published:** 2026-08-27

**Authors:** Yuye Ren, Jiao Ma, Wenya Hu, Lan Zhang, Xiaomin Wang

**Affiliations:** Department of Hematology, First Hospital of Shanxi Medical University, Taiyuan, China

**Keywords:** acute myeloid leukemia, bibliometric analysis, precision oncology, RNA sequencing, single-cell RNA sequencing

## Abstract

**Background:**

RNA sequencing (RNA-seq) has become an important tool for investigating the molecular heterogeneity of acute myeloid leukemia (AML); however, the global development and thematic evolution of this field remain inadequately characterized.

**Objective:**

To map the global landscape of AML RNA-seq research and identify major knowledge domains, emerging themes, and temporal changes in research priorities.

**Methods:**

Publications indexed in the Web of Science Core Collection and Scopus between January 1, 2007, and August 18, 2025, were retrieved. After database filtering, merging, and deduplication, 3,460 articles and reviews were included. CiteSpace, VOSviewer, the bibliometrix R package, and Microsoft Excel were used to analyze publication trends, collaboration networks, co-citation structures, keyword evolution, and citation bursts.

**Results:**

Publication output increased steadily, accelerating after 2014. China contributed the largest number of publications (*n* = 547, 15.8%), whereas the United States had the highest total citation count. Major publication outlets spanned hematology, oncology, genomics, and molecular biology. Co-citation analysis identified prominent themes involving next-generation sequencing, gene mutations, KMT2A rearrangements, epigenetic dysregulation, leukemia-initiating cells, drug resistance, biomarkers, T-cell biology, and single-cell sequencing. Earlier literature emphasized sequencing technologies, gene expression profiling, and molecular alterations, whereas recent publications show increasing representation of cellular heterogeneity, single-cell transcriptomics, drug resistance, biomarker applications, immune-related research, and computational interpretation.

**Conclusion:**

While molecular characterization remains foundational, AML RNA-seq research has broadened to encompass increasingly prominent cellular, functional, computational, and translational dimensions. This study provides a structured overview of the field; nevertheless, bibliometric prominence should not be interpreted as direct evidence of clinical utility.

## 1 Introduction

Acute myeloid leukemia (AML) is a biologically and clinically heterogeneous hematologic malignancy characterized by the clonal proliferation and impaired differentiation of myeloid progenitor cells, resulting in bone marrow failure and suppression of normal hematopoiesis. Its incidence increases markedly with age, particularly among individuals aged ≥65 years, and clinical outcomes vary substantially according to cytogenetic, molecular, and patient-related factors (1). Current treatment strategies include intensive chemotherapy, targeted therapies, and allogeneic hematopoietic stem cell transplantation in selected patients (2).

RNA sequencing (RNA-seq) has become an important tool for investigating the molecular complexity of AML. By enabling transcriptome-wide analysis, RNA-seq facilitates the characterization of gene expression patterns, molecular subtypes, fusion transcripts, regulatory programs, and potential therapeutic targets (3–6). Single-cell RNA sequencing has further increased analytical resolution by revealing leukemic cellular hierarchies, differentiation-associated states, and interactions between malignant and nonmalignant cell populations (7). Furthermore, RNA-seq-based studies have also identified potential therapeutic vulnerabilities related to NAMPT and detected rare molecular alterations, including the TMEM91:: TAL1 fusion (8, 9). Collectively, these developments have expanded AML transcriptomic research beyond molecular characterization toward biomarker discovery, risk stratification, treatment-response analysis, and translational precision-medicine research (3, 6, 10, 11).

Despite the rapid growth of AML RNA-seq research, its overall intellectual structure and thematic evolution remain insufficiently characterized. Existing studies span diverse areas, including molecular classification, treatment selection, risk assessment, bioinformatics, single-cell analysis, drug resistance, biomarker development, and clinical translation (12). For example, integrative transcriptomic and experimental studies have implicated the CBX4–HDAC5–CERS6 axis in sphingomyelin metabolic dysregulation in AML (13). However, the relationships between these research themes and changes in their prominence over time have not been systematically mapped. Previous bibliometric studies have examined AML as a broad research field or focused on specific subdomains, including targeted therapy, immunotherapy, autophagy, and single-cell sequencing (14–18). In contrast, the present study specifically maps RNA-seq research in AML across bulk transcriptomics, single-cell analysis, functional interpretation, and translational applications. By integrating publication trends, collaboration networks, co-citation structures, keyword evolution, and citation bursts using data from the Web of Science Core Collection and Scopus, this study aims to characterize both the intellectual structure and thematic evolution of the field.

Accordingly, this study presents a bibliometric analysis of AML RNA-seq research indexed between January 1, 2007, and August 18, 2025. Publication trends, country-, institution-, and author-level collaboration networks, journal and reference co-citation structures, keyword evolution, and citation bursts were analyzed to identify major research themes and their temporal development. Through the complementary use of CiteSpace, VOSviewer, and the bibliometrix R package, the study examined both the structural organization and changing research priorities of the field. The findings are intended to provide a systematic overview of AML RNA-seq research, clarify emerging biological and translational directions, and inform future investigations, while avoiding the assumption that bibliometric prominence directly indicates clinical utility.

## 2 Methods

### 2.1 Database and search strategy

**Figure 1:**
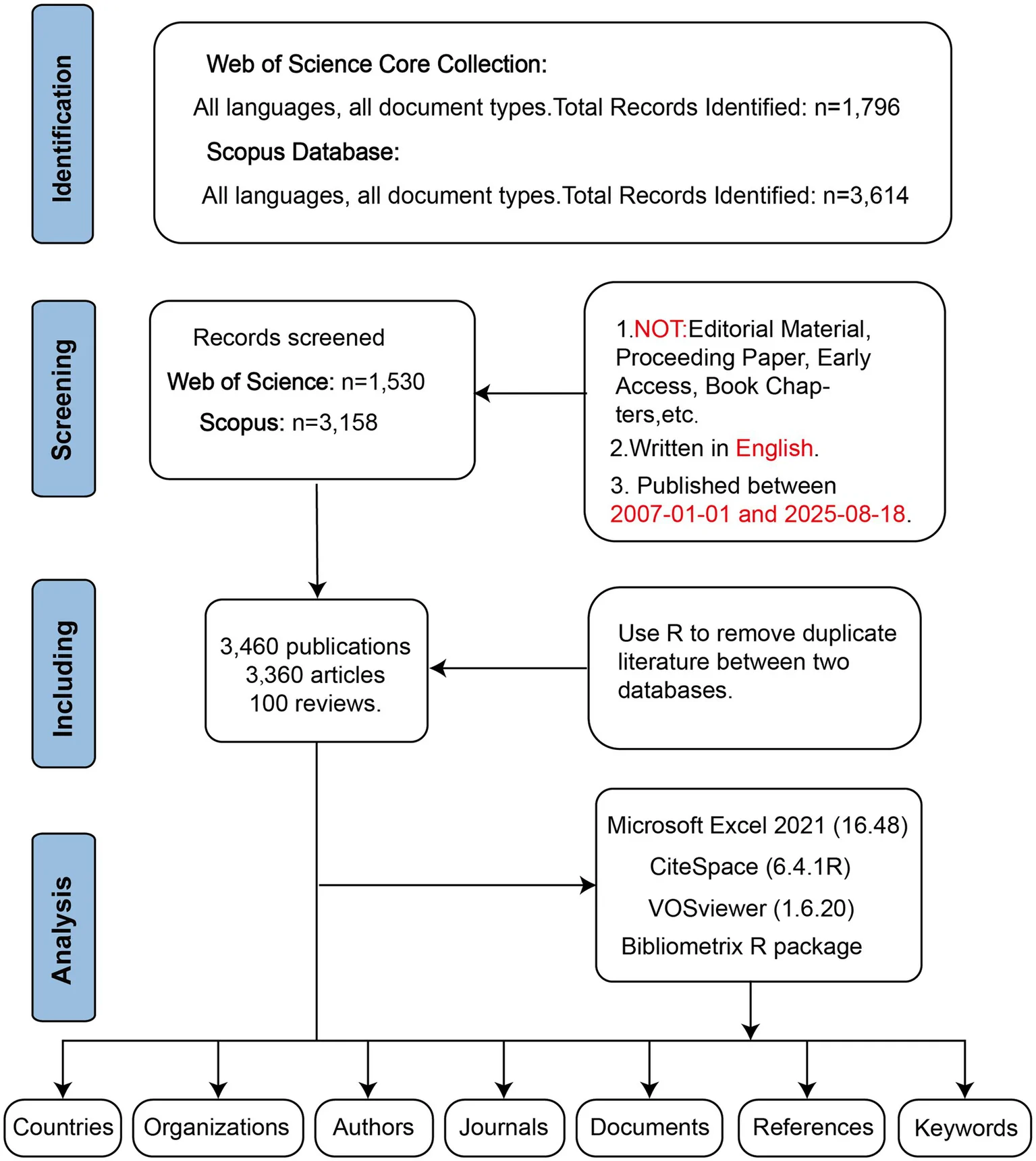
The flowchart for literature search, selection and analysis. TI, Title; AK, Author keywords; AB, Abstract.

As shown in Figure 1, data were retrieved from the Web of Science Core Collection (WoSCC) and Scopus on August 18, 2025 (19). WoSCC and Scopus were selected because they provide broad and complementary multidisciplinary coverage, standardized citation metadata, and data-export formats compatible with commonly used bibliometric software. The combined use of these databases was intended to enhance literature coverage and support reproducible citation and network analyses. In WoSCC, the topic search query was: TS = ((“RNA sequencing” OR “RNA seq” OR “RNA-seq” OR “Transcriptome sequencing” OR “Whole transcriptome sequencing” OR (“RNA expression profiling” OR “RNA profiling”) OR “Transcriptomic*” OR “mRNA sequencing” OR “single cell RNA sequencing” OR “scRNA seq” OR “scRNA-seq” OR “snRNA-seq” OR “spatial transcriptom*” OR “bulk RNA sequencing” OR (“next generation sequencing” AND (“RNA sequencing” OR “transcriptome”)) OR (“High-throughput sequencing” AND RNA) OR (“long-read RNA-seq” OR “Iso-Seq”))AND (“Acute myeloid leukemi*” OR “Acute myeloid leukaemi*” OR AML OR “Acute myelogenous leukemi*” OR “Acute myelogenous leukaemi*” OR “Acute myeloblastic leukemi*” OR “Acute myeloblastic leukaemi*” OR “Acute nonlymphocytic leukemi*” OR “Acute nonlymphocytic leukaemi*” OR ANLL)). In Scopus, the search query was:

TITLE-ABS-KEY ((“RNA sequencing” OR “RNA seq” OR “RNA-seq” OR “Transcriptome sequencing” OR “Whole transcriptome sequencing” OR “RNA expression profiling” OR “RNA profiling” OR “Transcriptomic*” OR “mRNA sequencing” OR “single cell RNA sequencing” OR “scRNA seq” OR “scRNA-seq” OR “snRNA-seq” OR “spatial transcriptom*” OR “bulk RNA sequencing” OR “High-Throughput Nucleotide Sequencing” OR “long-read RNA-seq” OR “Iso-Seq”) AND (“Acute myeloid leukemi*” OR “Acute myeloid leukaemi*” OR AML OR “Acute myelogenous leukemi*” OR “Acute myelogenous leukaemi*” OR “Acute myeloblastic leukemi*” OR “Acute myeloblastic leukaemi*” OR “Acute nonlymphocytic leukemi*” OR “Acute nonlymphocytic leukaemi*” OR ANLL)). The publication period was restricted to January 1, 2007, through August 18, 2025.

We restricted the results to English-language publications and retained only original articles and reviews. Document types, such as conference proceedings, corrections, early access items, news items, book chapters, retracted publications and retractions, reprints, biographical items, book reviews, meeting abstracts, editorial materials, and letters, were excluded. After applying the inclusion and exclusion criteria, 1,530 records were retrieved from the Web of Science Core Collection (WoSCC) and 3,158 records from Scopus. The records from both databases were subsequently merged, and duplicate publications were removed using R software. After deduplication, a total of 3,460 unique publications were retained for the final analysis.

### 2.2 Data processing

#### 2.2.1 CiteSpace

CiteSpace version 6.4. R1 (64-bit Advanced Edition) was used to perform co-occurrence analysis, co-citation cluster analysis, timeline visualization, and citation burst analysis (20). Knowledge maps of journals, references, and keywords were generated to visualize the intellectual structure and temporal evolution of the field.

#### 2.2.2 VOSviewer

Data were processed using VOSviewer 1.6.20, developed by CWTS at Leiden University (21). The full-counting method was applied, and item thresholds were defined separately for each analytical unit according to the frequency distribution of the corresponding items.

#### 2.2.3 Bibliometrix

The bibliometrix R package, developed by Massimo Aria and Corrado Cuccurullo, was used to analyze publication trends, source and author productivity, collaboration patterns, and bibliometric indicators, such as the number of publications, number of citations, *h*-index, and *g*-index (22).

#### 2.2.4 Other analytical tools

Microsoft Excel 2021 was used for preliminary data organization, data verification, and tabulation. The Online Analysis Platform of Literature Metrology was utilized as a supplementary tool to visualize selected bibliometric and citation indicators.

## 3 Results

### 3.1 Publication trends and general characteristics

A total of 3,460 publications on RNA sequencing (RNA-seq) in acute myeloid leukemia (AML), indexed between January 1, 2007, and August 18, 2025, were included in this bibliometric analysis (Supplementary Table S1). Because the literature search was completed in August 2025, the publication count for 2025 represents partial-year data and was not directly compared with the complete annual outputs of previous years.

**Figure 2:**
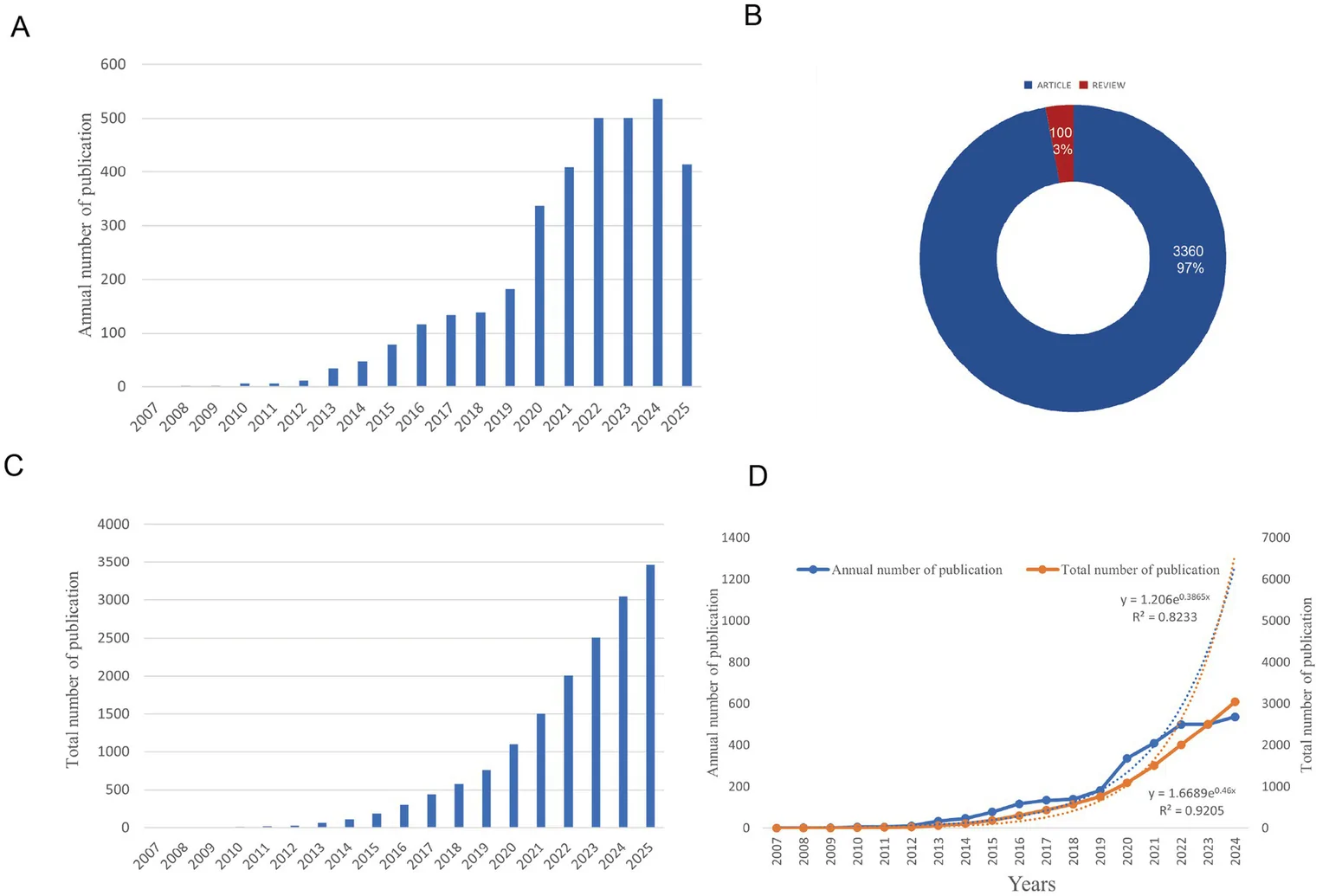
Publication trends in RNA sequencing research on acute myeloid leukemia (2007–2025). **(A)** Annual number of publications from 2007 to 2025. **(B)** Distribution of document types. **(C)** Cumulative number of publications over time. **(D)** Price’s Law growth curve showing the relationship between annual and cumulative publications with exponential fitting.

Annual publication output increased gradually during the early study period and accelerated markedly after 2014 (Figure 2A). Correspondingly, the cumulative publication curve became progressively steeper over time (Figure 2C). Exponential fitting based on complete-year data from 2007 to 2024 demonstrated an approximately exponential growth pattern (Figure 2D).

Original research articles accounted for 3,360 publications (97.0%), whereas reviews accounted for 100 publications (3.0%) (Figure 2B). A total of 22,339 authors contributed to the included literature, with a mean of 11.4 authors per document, indicating that publications in this field were predominantly produced by relatively large research teams.

### 3.2 Country-level contributions and international collaboration

**Figure 3:**
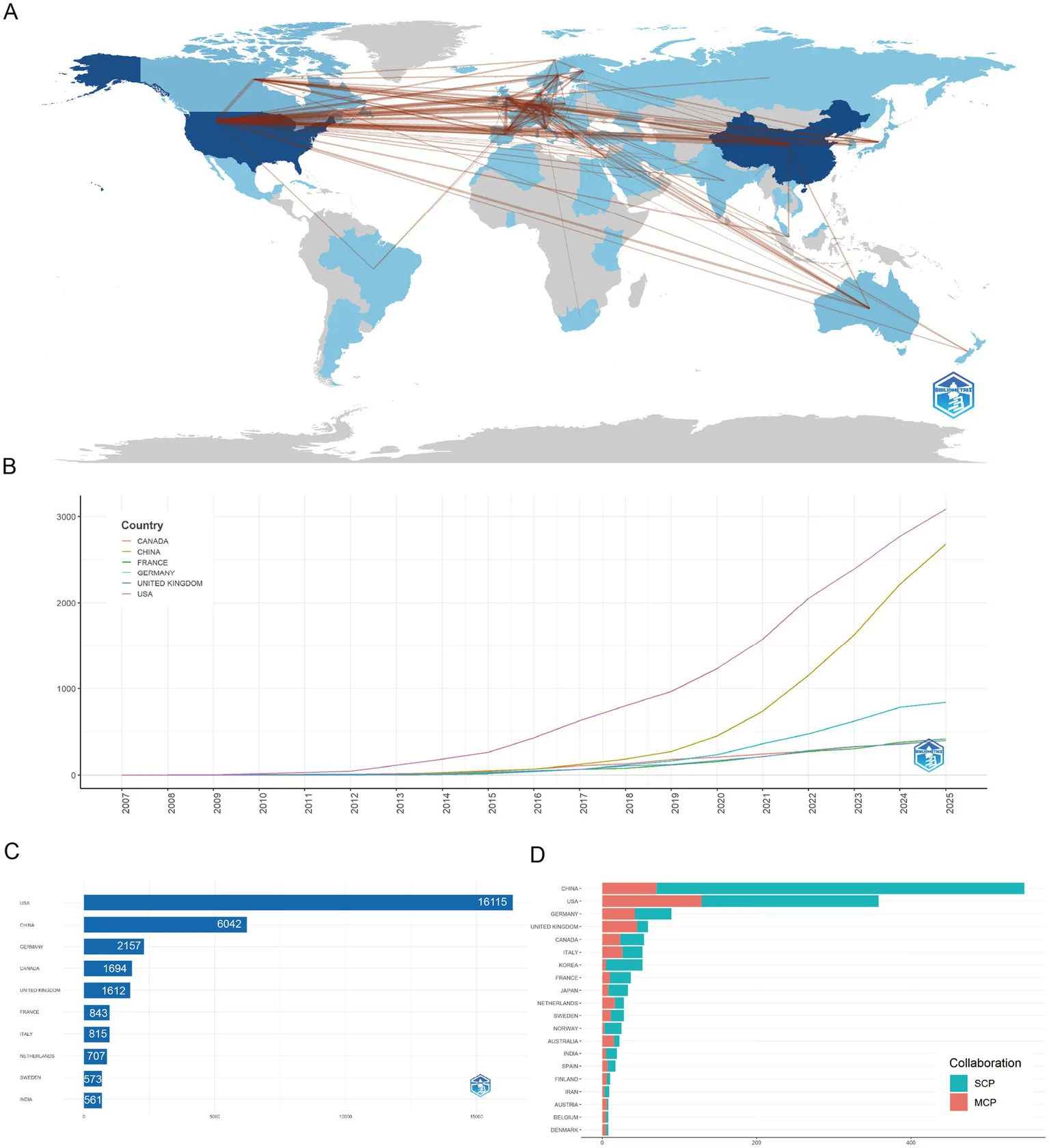
Global trends and collaboration networks in RNA sequencing research on acute myeloid leukemia. **(A)** Global collaboration map of co-authorship networks. Darker blue shades indicate higher publication output, and lines represent international collaborative ties. **(B)** Country publication trends over time. The *x*-axis represents years, and the *y*-axis shows the cumulative number of publications. **(C)** Total citation counts across countries. **(D)** The top 10 most productive countries and their collaboration patterns (Single-Country Publications and Multiple-Country Publications). SCP, Single-Country Publications; MCP, Multiple-Country Publications.

The geographic distribution and international collaboration network demonstrated that AML RNA-seq research was concentrated primarily in North America, Europe, and Asia (Figure 3A). Based on corresponding-author affiliations, China ranked first in publication output, with 547 publications accounting for 15.8% of the total, followed by the United States with 358 publications accounting for 10.3% (Supplementary Table S2). By contrast, the United States achieved the highest accumulated citation count (Figure 3C), indicating that country rankings differed between publication output and citation visibility.

The cumulative country-production trajectories revealed a marked increase in China’s publication output, particularly after 2015, whereas the United States maintained sustained growth throughout the study period (Figure 3B). Germany, the United Kingdom, Italy, and France were also among the major contributing countries. Figure 3B presents cumulative production based on country-level affiliation records, whereas Supplementary Table S2 reports countries according to corresponding-author affiliations; consequently, their absolute publication counts represent different statistical indicators.

Analysis of single-country publications (SCPs) and multiple-country publications (MCPs) highlighted substantial variation in international collaboration patterns. China had an MCP proportion of 12.8%, whereas the United Kingdom, Australia, and the Netherlands had MCP proportions of 76.3, 68.2, and 57.1%, respectively (Figure 3D; Supplementary Table S2). Country rankings therefore varied across publication output, accumulated citations, and international collaboration tendencies.

### 3.3 Institutional contributions and collaboration

**Figure 4:**
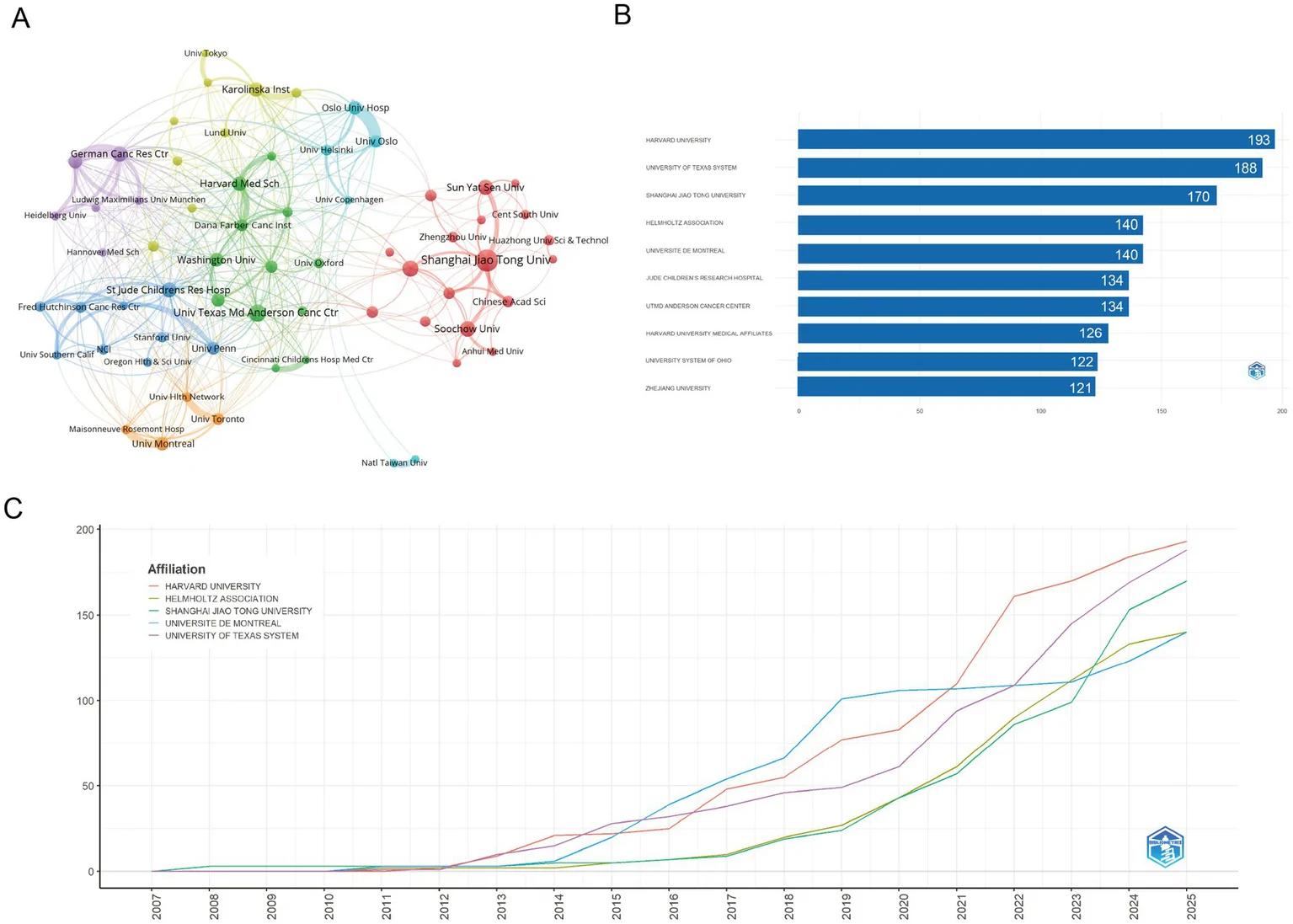
Institutional contributions and collaboration networks in RNA sequencing research on acute myeloid leukemia. **(A)** Institutional collaboration network based on co-authorship. Nodes represent institutions, with sizes reflecting publication output and links indicating collaboration relationships. Colors denote institutional clusters. **(B)** Top affiliations ranked by publication volume. **(C)** Publication trends of leading institutions over time. The *x*-axis denotes years, and the *y*-axis shows publication counts.

The institutional collaboration network comprised multiple interconnected clusters (Figure 4A). Large and highly connected nodes included Harvard University, Dana-Farber Cancer Institute, the Broad Institute, and several other major biomedical research centers. Based on publication volume, Harvard University, the University of Texas System, and Shanghai Jiao Tong University were among the leading affiliations (Figure 4B).

Chinese institutions, including Shanghai Jiao Tong University, the Chinese Academy of Sciences, and Peking University, showed increasing publication activity during the later years of the study period. The cumulative publication trajectories of leading institutions are presented in Figure 4C. Overall, the network showed extensive collaboration among institutions from different geographic and organizational clusters.

### 3.4 Authors and co-cited authors

**Figure 5:**
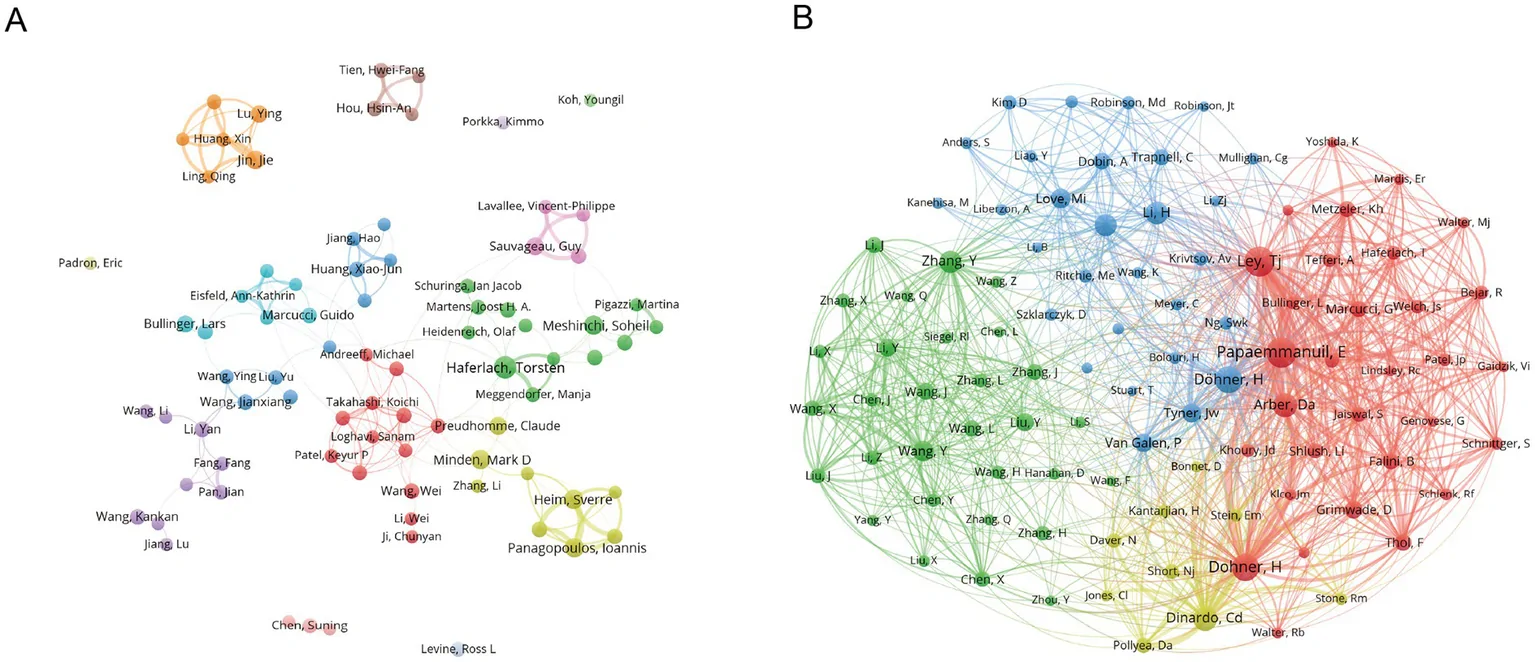
Co-authorship and co-citation networks of authors in RNA sequencing research on acute myeloid leukemia. **(A)** Co-authorship network. Nodes represent authors, with sizes reflecting publication count and links indicating co-authorship relationships. Colors denote collaboration clusters. **(B)** Co-citation network of authors. Nodes represent authors, and links indicate co-citation relationships. Edge thickness reflects the frequency of co-citation. Colors indicate different research clusters.

The author co-authorship network comprised several interconnected research communities involving investigators from different institutions and countries (Figure 5A). Among the five authors with the highest dataset-specific h-indices, Liu Y and Wang J each had an *h*-index of 22. Wang Y had the largest publication output, with 99 articles, followed by Wang J with 97 articles, and Liu Y with 94 articles. Bullinger L and Zhang J also showed substantial publication output and citation performance (Supplementary Table S3).

The author co-citation network identified frequently co-cited researchers, including Ding L, Tyner JW, and Van Galen P (Figure 5B). These authors were associated with highly cited studies on clonal evolution, functional genomics, and single-cell characterization of AML. Productive authors and frequently co-cited authors showed only partial overlap, reflecting the distinction between publication productivity and influence within the cited literature.

### 3.5 Journals and co-cited journals

**Figure 6:**
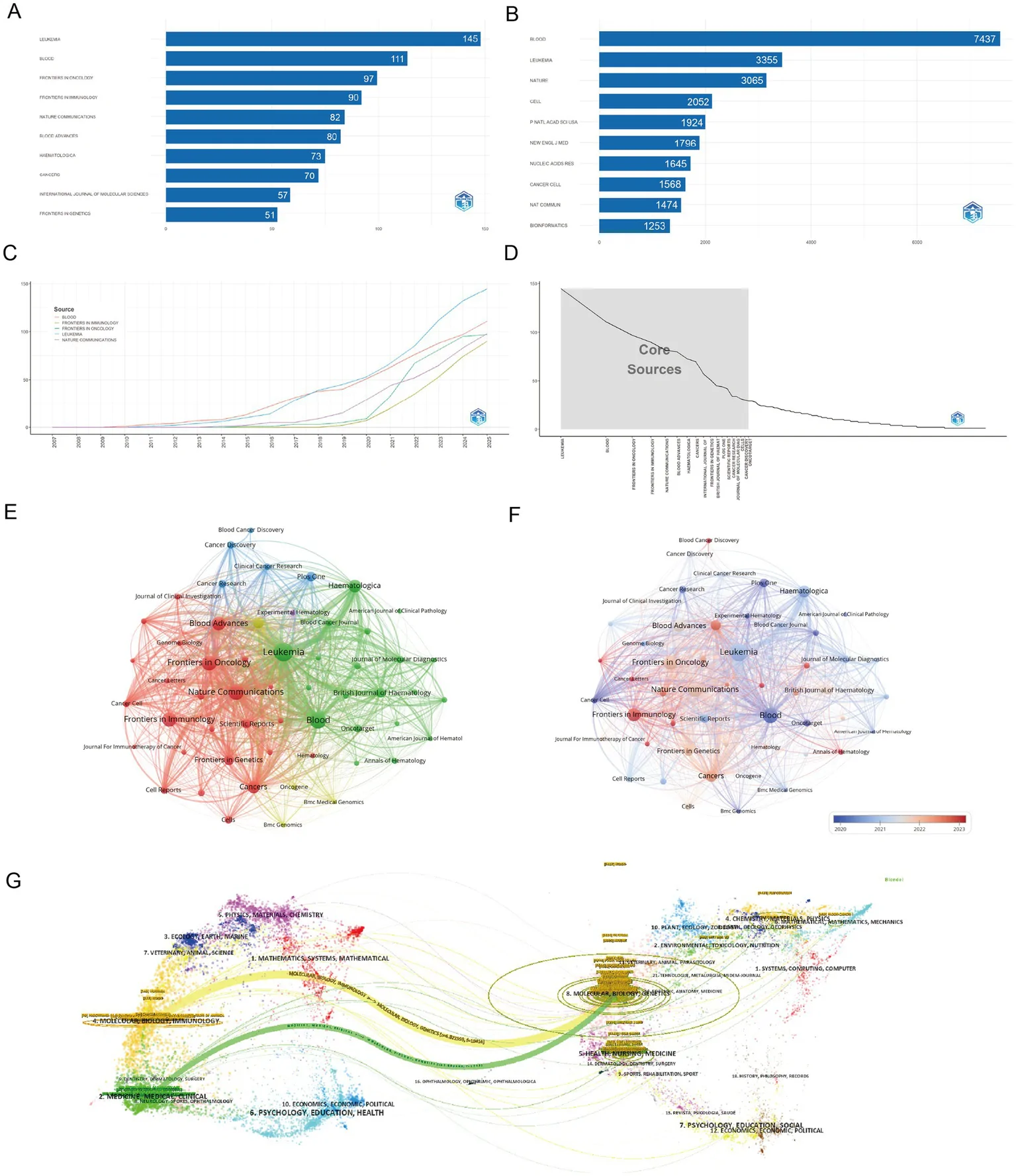
Journal analysis and citation networks in RNA sequencing research on acute myeloid leukemia. **(A)** Top journals ranked by publication count. **(B)** Top 10 journals ranked by local citation frequency within the retrieved dataset. **(C)** Cumulative publication trajectories of the five leading journals. **(D)** Bradford’s Law analysis showing the distribution of core journals. **(E)** Journal co-citation network. Node size reflects co-citation frequency, links represent co-citation relationships, and colors denote clusters of frequently co-cited journals. **(F)** Overlay visualization of the journal citation network. Node color indicates the average publication year. **(G)** Dual-map overlay showing citation pathways between citing and cited journal domains.

AML RNA-seq studies were published primarily in journals covering hematology, oncology, genomics, and molecular biology (Figure 6). *Leukemia* had the highest publication volume, with 145 articles, followed by *Blood* with 111 articles, *Frontiers in Oncology* with 97 articles, *Nature Communications* with 82 articles, and *Blood Advances* with 80 articles (Figure 6A; Supplementary Table S4).

Within-dataset journal co-citation analysis demonstrated that *Blood* had the highest local citation frequency, with 7,437 citations, followed by *Leukemia* with 3,555 and *Nature* with 3,065 (Figure 6B). Based on the aggregated global citations received by the included publications in each journal, *Blood* ranked first with 6,000 citations, followed by *Nature Communications* with 3,957 and *Leukemia* with 3,839 (Supplementary Table S4). Journal rankings therefore differed across publication output, local citation frequency, and accumulated global citations.

The cumulative publication trajectories of the five leading journals are presented in Figure 6C. Bradford’s law analysis demonstrated that publications were concentrated within a relatively small group of specialized journals (Figure 6D). The journal co-citation network revealed close relationships among major biomedical journals, including *Nature*, *Cell*, *Cancer Discovery*, *Blood*, and *Leukemia* (Figures 6E,F). The dual-map overlay showed major citation paths connecting molecular, biological, and genetic source journals with oncology- and clinically oriented target journals (Figure 6G).

### 3.6 Highly cited references, co-citation structure, and citation bursts

Global citation analysis identified the most highly cited publications within the retrieved dataset. The 10 publications with the highest global citation counts are presented in Supplementary Table S5. Ding et al. (23), in their seminal article entitled “*Clonal evolution in relapsed acute myeloid leukaemia revealed by whole-genome sequencing*,” was the most highly cited publication, receiving 1,698 citations. Other highly cited publications included the study by Tyner et al. (6) on the functional genomic landscape of AML, the single-cell RNA-sequencing study by Van Galen et al. (19), and the measurable residual disease study by Jongen-Lavrencic et al. (24). These publications addressed clonal evolution, functional genomics, cellular hierarchies, and longitudinal molecular monitoring, respectively. Broader topics in cancer genomics, RNA regulation, sequencing technologies, and pan-cancer analysis were also represented among the most highly cited records.

**Figure 7:**
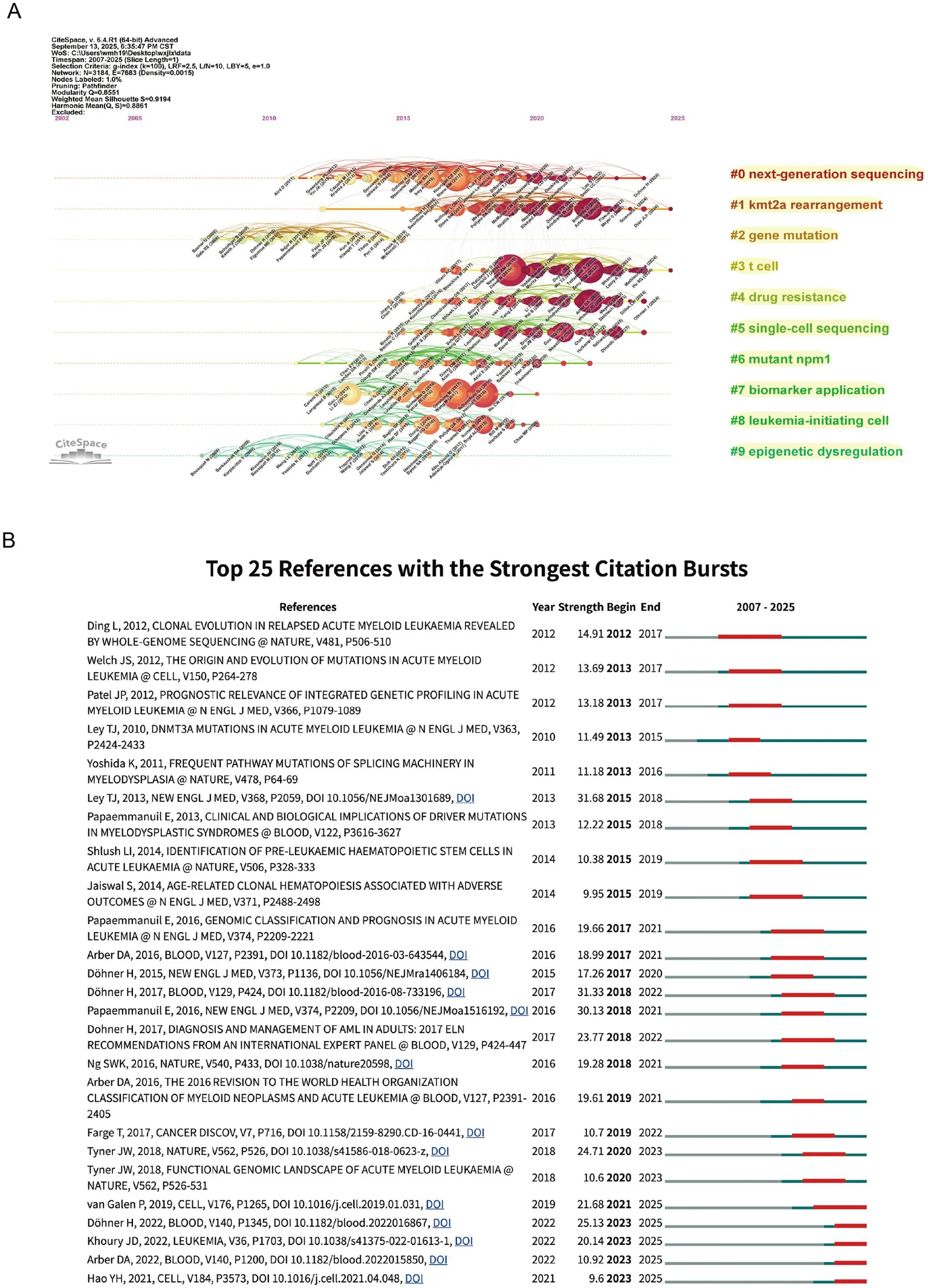
Reference co-citation network and top 25 references with the strongest citation bursts in RNA sequencing research on acute myeloid leukemia. **(A)** Timeline visualization of reference co-citation clusters. Each horizontal line represents an algorithmically generated cluster, and nodes represent cited references over time. Node size reflects citation frequency, and node colors indicate different time slices. The network had a modularity *Q* value of 0.8561 and a weighted mean silhouette *S* value of 0.6194. **(B)** The 25 references with the strongest citation bursts. Blue lines represent the entire observation period, whereas red segments indicate periods of markedly increased citation activity.

Reference co-citation analysis was conducted to characterize the intellectual structure of AML RNA-seq research. The network yielded a modularity *Q* value of 0.8561 and a weighted mean silhouette *S* value of 0.6194, indicating a well-defined modular structure with moderate cluster consistency. The timeline visualization identified 10 algorithmically generated clusters labeled “next-generation sequencing,” “KMT2A rearrangement,” “gene mutation,” “T cell,” “drug resistance,” “single-cell sequencing,” “mutant NPM1,” “biomarker application,” “leukemia-initiating cell,” and “epigenetic dysregulation” (Figure 7A).

The clusters overlapped temporally but differed in their periods of citation prominence. Clusters related to sequencing technologies and gene mutations were more frequently represented during earlier periods, whereas clusters associated with drug resistance, leukemia-initiating cells, biomarker applications, T-cell biology, epigenetic dysregulation, and single-cell sequencing gained prominence during later periods.

Reference citation burst analysis identified publications that experienced marked surges in citation activity during specific periods (Figure 7B). The 25 references with the strongest citation bursts addressed genomic characterization, molecular classification, functional genomics, measurable residual disease, and single-cell analysis. Foundational studies of clonal evolution and functional genomics demonstrated sustained citation bursts, whereas more recent burst references focused on cellular hierarchies and single-cell heterogeneity.

### 3.7 Keyword structure and temporal evolution of research hotspots

**Figure 8:**
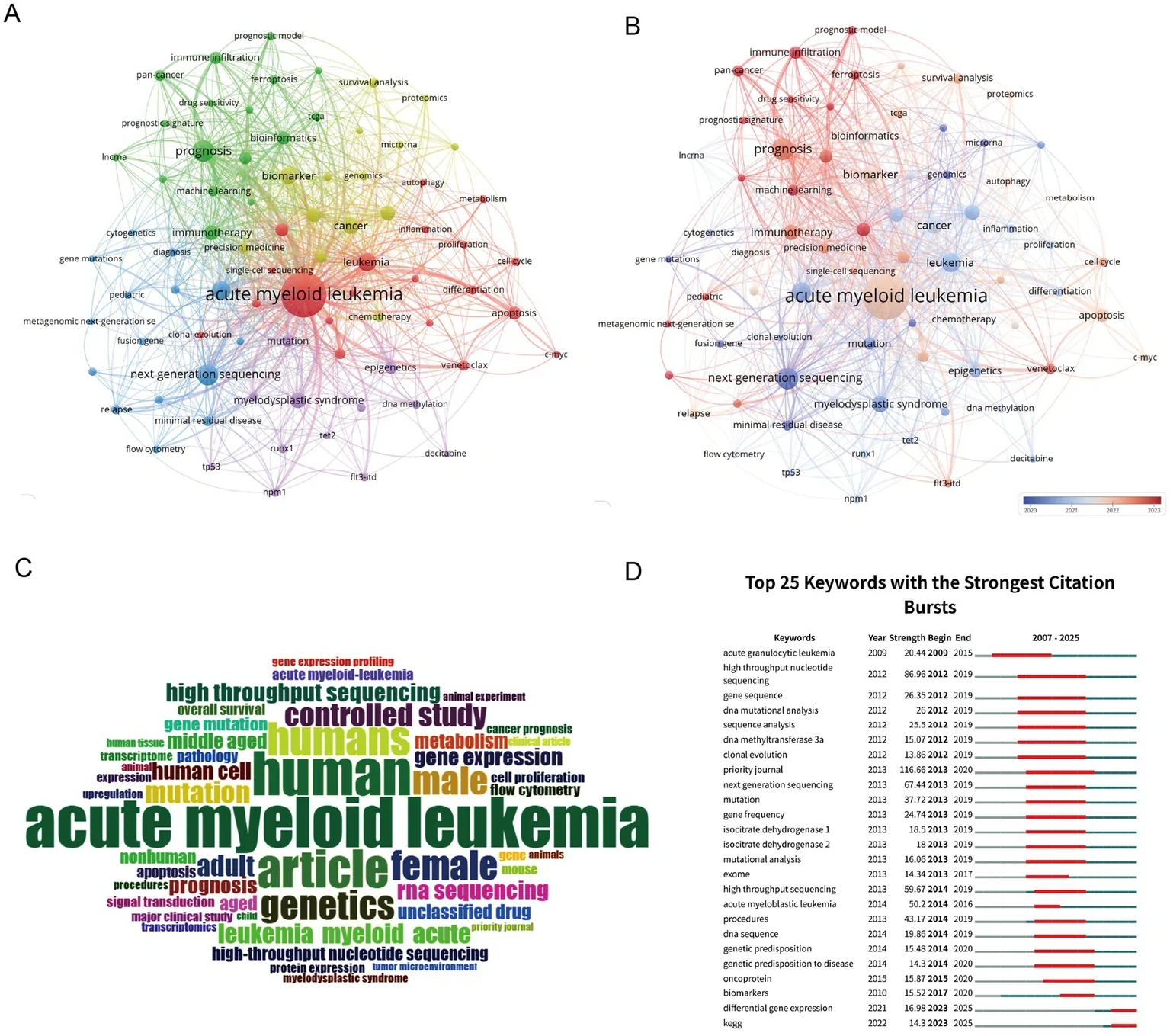
Keyword analysis in RNA sequencing research on acute myeloid leukemia. **(A)** Keyword co-occurrence network. Node size reflects occurrence frequency, links indicate co-occurrence relationships, and colors denote thematic clusters. **(B)** Temporal overlay visualization of keyword co-occurrence. Node color indicates the average publication year, with blue representing earlier topics and red representing more recent topics. **(C)** Keywords Plus word cloud. Word size reflects term frequency. **(D)** The 25 keywords with the strongest bursts. Red segments indicate periods during which the occurrence frequency of each keyword increased rapidly.

Keyword co-occurrence mapping, temporal overlay visualization, keywords plus analysis, and burst detection were used to characterize the thematic structure and temporal development of AML RNA-seq research (Figure 8). The keyword co-occurrence network comprised several interconnected thematic groups, with “acute myeloid leukemia,” “RNA sequencing,” “gene expression,” and “mutation” forming prominent nodes (Figure 8A).

The network broadly encompassed five thematic areas: genomic and transcriptomic characterization; molecular alterations and epigenetic regulation; prognosis and biomarker research; computational and single-cell analysis; and immune- and treatment-related research. Representative terms included “NPM1,” “FLT3-ITD,” “DNA methylation,” “machine learning,” “immune infiltration,” “immunotherapy,” “drug sensitivity,” and “venetoclax.”

The temporal overlay visualization demonstrated differences in the average publication years associated with individual keywords (Figure 8B). Keywords related to sequencing technologies, genomic diagnosis, gene mutations, fusion genes, and clonal evolution generally exhibited earlier average publication years. In contrast, keywords related to prognosis, drug sensitivity, immune infiltration, machine learning, and single-cell sequencing were associated with more recent publications.

Keywords plus analysis was dominated by broad indexing terms, including “human,” “article,” “male,” “female,” “controlled study,” “genetics,” and “acute myeloid leukemia” (Figure 8C). Because these terms offered limited thematic specificity, the keywords plus word cloud was interpreted descriptively rather than used independently to identify research hotspots.

Keyword citation burst analysis identified distinct periods of rapidly increasing term frequency (Figure 8D). Earlier bursts were dominated by sequencing- and mutation-related terms, including “high-throughput nucleotide sequencing,” “gene sequence,” “DNA mutational analysis,” “DNMT3A,” “next-generation sequencing,” “mutation,” “IDH1,” and “IDH2.” The term “biomarkers” showed a burst from 2017 to 2020. More recently, “differential gene expression” exhibited a burst from 2021 to 2025, whereas “Kyoto encyclopedia of genes and genomes (KEGG)” demonstrated a burst from 2022 to 2025. Overall, the keyword analyses displayed temporal overlap between established genomic themes and emerging computational, single-cell, immune-related, treatment-related, and pathway-oriented topics.

## 4 Discussion

By integrating keyword co-occurrence and temporal mapping, burst detection of keywords and cited references, and reference co-citation analysis, this study delineates the evolving intellectual structure of RNA sequencing research in AML. The bibliometric results indicate three overlapping transitions in research emphasis: an expansion from molecular characterization toward functional and prognostic stratification, a broadening from individual genes and bulk expression profiles toward pathway-level and single-cell analyses, and growing interest in clinically oriented questions such as biomarker development, treatment response, and disease monitoring. Early research was dominated by sequencing-, mutation-, and gene-expression-related topics, with epigenetic regulation forming an important associated theme, whereas more recent studies increasingly encompass cellular heterogeneity, drug resistance, leukemia stem-cell biology, and computational interpretation. These shifts are reflected in the reference co-citation structure and in the temporal distribution and bursts of keywords shown in Figures 7, 8.

These patterns should not be interpreted as a linear replacement of earlier topics by newer ones. Molecular characterization remains fundamental to AML research, whereas subsequent approaches have expanded its analytical scope and biological resolution. Nor do the observed trends demonstrate that RNA-seq has achieved routine implementation in clinical AML management. Bibliometric methods identify the prominence, relationships, and temporal development of research topics, but they do not directly establish diagnostic accuracy, therapeutic efficacy, or clinical utility. The representative studies discussed below are consequently cited to provide biological and clinical context for the bibliometric findings rather than to provide an independent systematic assessment of clinical evidence.

### 4.1 Evolution from molecular characterization to functional stratification

The early keyword timeline and citation burst analysis were dominated by terms related to DNA sequencing, mutations, and gene expression, whereas the reference co-citation network comprised algorithmically generated clusters labeled “next-generation sequencing,” “gene mutation,” and “epigenetic dysregulation” (Figures 7A, 8B,D). Together, these findings indicate that early AML transcriptomic research primarily focused on defining molecular abnormalities associated with leukemogenesis and disease heterogeneity. These early studies subsequently provided a basis for investigations into clonal evolution and relapse.

Because transcriptional states in AML are commonly interpreted in relation to underlying genomic lesions, genomic studies provided an important conceptual foundation for subsequent RNA-seq research (25). Whole-genome sequencing of paired diagnostic and relapse samples demonstrated that AML relapse may arise through the continued evolution of the founding clone or the expansion of a preexisting subclone under treatment-associated selection pressure (23). Integrated genomic and epigenomic profiling subsequently identified recurrent alterations involving transcription factors, epigenetic regulators, signaling pathways, spliceosome components, and tumor suppressors, thereby establishing a molecular framework for interpreting transcriptional heterogeneity in AML (25). Although these studies were not based exclusively on RNA-seq, they clarified the genomic context within which RNA-level variation could be understood.

The concurrent prominence of mutation-, methylation-, and epigenetic-regulation-related topics further suggests that the field gradually moved beyond mutation cataloging toward examining the functional consequences of genomic lesions. Representative studies of DNMT3A- and IDH1/2-mutated AML illustrate this development. DNMT3A-mutated hematopoietic stem cells were shown to precede overt AML, persist during remission, and provide a potential reservoir for subsequent leukemic evolution (26). AML harboring IDH1 or IDH2 mutations was associated with a characteristic DNA hypermethylation phenotype, illustrating how mutation-associated metabolic changes may alter differentiation and transcriptional regulation (27). These studies provide biological context for the close interrelationships among mutation, methylation, and gene-expression themes identified in the bibliometric analysis.

As the field developed, attention increasingly shifted from isolated abnormalities toward integrated molecular states. The growing representation of prognosis-, biomarker-, molecular-signature-, and leukemia stem-cell-related topics in the co-citation and keyword networks is consistent with this change in emphasis (Figures 7A, 8A,B). Large-scale molecular classification demonstrated that the prognostic effect of an individual mutation depends substantially on its co-occurrence with other driver lesions, supporting a move from single-gene interpretation toward integrated molecular classification (28). This framework also provided a basis for interpreting transcriptomic profiles as composite representations of genomic, epigenetic, and cellular states.

Transcriptome-derived signatures represent a further step toward functional and prognostic stratification. The 17-gene leukemia stem-cell score was developed to quantify stemness-associated expression patterns and identify patients with adverse outcomes and limited benefit from standard therapy (10). This study illustrates how gene expression data can be transformed from descriptive measurements into potentially informative risk-stratification models. Nevertheless, such signatures require validation in independent cohorts and assessment of their incremental value beyond established cytogenetic and molecular risk systems.

Overall, a major shift identified in the bibliometric landscape was not merely an increase in sequencing activity. Rather, it involved an expansion from documenting molecular abnormalities toward interpreting how interacting genomic, epigenetic, and transcriptional states relate to AML phenotype and prognosis.

### 4.2 Expansion toward pathway-level and single-cell analysis

Another major development in AML RNA-seq research unfolded along two related but distinct analytical directions. The first involved a shift from individual differentially expressed genes toward pathways, regulatory networks, and integrated molecular programs. The keyword co-occurrence structure and temporal mapping, together with recent citation bursts for “differential gene expression” and “KEGG,” indicate increasing attention to functional and pathway-level interpretation (Figures 8A,B,D).

A broader manifestation of this systems-level approach is the integration of genomic, transcriptomic, clinical, and functional drug-response data. The Beat AML program demonstrated that ex vivo drug-response patterns can be associated with particular combinations of genetic and transcriptional features rather than with individual mutations alone (6, 11). Such studies broadened the analytical question from identifying altered genes to examining how interacting molecular programs relate to cellular states and therapeutic response. The growing prominence of bioinformatics and pathway analysis is consequently consistent with both methodological expansion and increasing interest in systems-level interpretation of AML biology.

The second direction involved a shift in analytical resolution from bulk cell populations to individual cellular states. The appearance of the reference co-citation cluster labeled “single-cell sequencing,” together with the increasing prominence of single-cell-related terms in the keyword timeline, indicates that cellular heterogeneity has become an important component of recent AML transcriptomic research (Figures 7A,B).

Bulk RNA-seq provides an averaged expression profile across heterogeneous cell populations and may obscure rare leukemic subpopulations, distinct differentiation states, and nonmalignant cells within the sample. Single-cell RNA sequencing partially addresses this limitation by resolving transcriptional variation at cellular resolution (29). Van Galen et al. combined single-cell transcriptomics with genotyping and identified malignant cell populations resembling different stages of normal myeloid differentiation. Primitive AML cells displayed transcriptional programs associated with stemness and myeloid priming (30). An independent single-cell investigation similarly demonstrated substantial variation in AML cellular hierarchies and identified differentiation-associated transcriptional states across patient samples (31). These studies provide biological context for the prominence of single-cell sequencing, leukemia stem-cell biology, and cellular heterogeneity in the bibliometric results.

Single-cell approaches have also extended the focus beyond leukemia-intrinsic abnormalities to nonmalignant cellular compartments and microenvironmental interactions (30, 32). Single-cell characterization of the mouse bone marrow stroma identified diverse stromal populations and demonstrated that emerging AML impaired mesenchymal osteogenic differentiation and reduced the expression of regulatory molecules required for normal hematopoiesis (32). Other transcriptomic studies identified immune dysfunction signatures associated with adverse molecular features and clinical outcomes, suggesting that immune-cell states constitute an additional dimension of AML heterogeneity (33). These findings are consistent with increasing attention regarding the contribution of the bone marrow microenvironment to disease biology.

Thematic evolution can consequently be understood as an expansion from individual genes to pathways and regulatory programs, from population-averaged profiles to heterogeneous cellular states, and from leukemia-intrinsic abnormalities to interactions between malignant and nonmalignant compartments.

However, increased analytical resolution introduces additional sources of variability. Differences in specimen source, sample processing, cell isolation, sequencing depth, cell annotation, batch correction, and computational pipelines can substantially impact the cellular classifications and molecular signatures obtained. These methodological variations currently limit direct comparison and integration across cohorts.

### 4.3 Emerging translational implications

The co-citation clusters labeled “drug resistance” and “biomarker application” indicate sustained attention to clinically oriented questions. The increasing occurrence of prognosis-, molecular-stratification-, and treatment-response-related keywords further suggests an effort to connect molecular findings with clinical challenges (Figures 7A, 8A,B). These patterns support growing translational interest, although they do not by themselves demonstrate clinical implementation.

One translational direction involves using transcriptomic information to complement cytogenetic and genomic classification (10, 28). Patients with the same driver alteration may exhibit different transcriptional programs, differentiation states, microenvironmental interactions, and drug-response profiles (6, 11). Expression-based signatures may therefore capture biological variation that is not fully represented by mutation status alone. Transcriptome-derived models such as LSC17 illustrate the potential of expression-based information to complement established risk stratification (10). Integrated analyses of genomic, transcriptomic, clinical, and drug-response data have also demonstrated that broader molecular and cellular contexts can explain variation in ex vivo drug sensitivity that is not captured by individual mutations (11).

A second translational direction concerns treatment response and resistance. The presence of a drug resistance-related co-citation cluster reflects sustained research interest in the biological basis of therapeutic failure. A representative study illustrating this broader theme demonstrated that monocytic AML subpopulations exhibit reduced dependence on BCL-2 and increased resistance to venetoclax-based therapy (34). This association indicates that differentiation-associated transcriptional states can contribute to variation in treatment response. Such findings support the identification of biologically defined subgroups and the generation of hypotheses for combination therapy, though they do not establish validated predictive biomarkers for routine treatment selection.

A third translational direction involves longitudinal molecular assessment. Molecular assessment of measurable residual disease (MRD) holds substantial prognostic value in AML. Persistent disease-associated mutations during remission are associated with an increased risk of relapse and inferior survival (24). However, established MRD approaches predominantly rely on targeted mutation detection, fusion-transcript quantification, or multiparameter flow cytometry rather than unbiased whole-transcriptome RNA-seq (35). The prominence of MRD-related references in the co-citation structure should consequently be interpreted as evidence of growing interest in longitudinal molecular monitoring, not as proof that whole-transcriptome RNA-seq has been validated for routine MRD assessment. At present, RNA-seq is better regarded as a complementary research approach for investigating residual cellular states, resistance-associated programs, and mechanisms of disease evolution (36, 37).

Several methodological and clinical barriers limit the translation of RNA-seq-derived signatures. Many candidate biomarkers are derived from retrospective or limited cohorts and require independent external validation before clinical implementation (38). Differences in specimen processing, RNA quality, library preparation, sequencing depth, normalization, and analytical pipelines can diminish cross-study reproducibility (39). Bulk RNA-seq signatures may partly reflect variation in cellular composition rather than leukemia cell-intrinsic biology (40), whereas single-cell assays remain technically demanding, resource-intensive, and difficult to standardize for routine clinical workflows (41).

Clinical adoption also requires rigor beyond mere statistical association. Candidate models must demonstrate analytical robustness, reproducibility across laboratories and populations, predefined and interpretable thresholds, and added value beyond established cytogenetic, genomic, and immunophenotypic systems. Prospective studies are needed to determine whether transcriptomic information changes clinical decisions or improves patient outcomes. Thus, the bibliometric findings support an emerging but still exploratory translational trajectory involving prognosis, treatment response, resistance, and disease monitoring.

### 4.4 Future directions and unresolved challenges

The present bibliometric analysis identified single-cell sequencing, cellular heterogeneity, bioinformatics, molecular biomarkers, differential gene expression, and pathway-level interpretation as prominent recent themes (Figures 7A, 8B,D). These themes are poised to influence the near-term research agenda, although publication and citation patterns alone cannot determine their long-term scientific importance or clinical feasibility.

Further integration of transcriptomic data with genomic, epigenomic, proteomic, metabolic, and functional measurements could help clarify relationships among molecular lesions, cellular states, leukemic evolution, and treatment response. Multimodal single-cell approaches such as DOGMA-seq offer the potential to jointly characterize gene expression, chromatin accessibility, and cell-surface protein levels in AML (42, 43). Their reproducibility, feasibility, and added value over more accessible assays, however, require rigorous evaluation in representative patient cohorts.

Machine learning emerged as a recent computational theme in the keyword analysis and can facilitate the integration of high-dimensional molecular and clinical variables (44). However, its application remains sensitive to sample size, data harmonization, model interpretability, overfitting, and external validation (45, 46). Broader artificial-intelligence applications and spatial transcriptomics were not identified as dominant themes in the present analysis, but could represent potential extensions of the current research trajectory. Spatial transcriptomics can preserve anatomical information relevant to leukemia–microenvironment interactions, although its application is constrained by tissue availability, sample preservation, spatial resolution, platform variability, and cross-study comparability (47, 48).

Methodological harmonization will be central to progress across these areas. Standardized sample collection and processing, transparent reporting of sequencing and computational procedures, appropriate correction for batch and cellular-composition effects, and shared reference datasets will improve cross-study comparability (49, 50). Future research must also distinguish clearly among analytical performance, biological validity, and clinical utility. A signature can be statistically reproducible and biologically informative without improving treatment selection or patient outcomes (51).

Overall, the bibliometric patterns demonstrate that AML RNA-seq research has broadened in biological scope, analytical resolution, and translational orientation. Converting these developments into clinically actionable tools will require methodological standardization, independent validation, and the prospective demonstration of added clinical value.

## 5 Conclusion

This bibliometric study provides a comprehensive overview of AML RNA-seq research from January 2007 to August 2025. Publication trends, reference co-citation networks, keyword evolution, and citation bursts indicate that molecular characterization has remained a central theme, whereas functional and prognostic stratification, pathway-level interpretation, cellular heterogeneity, computational analysis, and clinically oriented research have gained increasing prominence.

These patterns reflect an expansion of research priorities rather than the routine clinical implementation of RNA-seq. Translating transcriptomic findings into clinically actionable tools will require methodological harmonization, independent validation, prospective assessment, and the demonstration of added value beyond established diagnostic and risk-stratification approaches. RNA sequencing is consequently likely to remain an important platform for advancing AML biology and supporting future translational research.

## 6 Limitations

Several limitations should be considered when interpreting these findings. Although both the Web of Science Core Collection and Scopus were searched, differences in database coverage and indexing practices may have led to the omission of relevant studies. Restricting the search to English-language publications may also have narrowed literature coverage. In addition, because the search was completed on August 18, 2025, publication data for 2025 were incomplete, and more recent studies possessed shorter citation windows.

Bibliometric indicators reflect scholarly visibility rather than methodological quality or clinical significance. Citation counts can be influenced by publication age, self-citation, journal visibility, and topic popularity. Author and institutional analyses may also be affected by incomplete name disambiguation, whereas co-citation clustering and burst detection are sensitive to software settings and analytical parameters.

Finally, the biological and clinical interpretations presented in the Discussion represent contextual explanations supported by representative studies rather than conclusions derived directly from bibliometric indicators. Potential future directions, including broader artificial intelligence applications, spatial transcriptomics, and multi-omics integration, therefore require further validation and prospective investigation.

## Data Availability

The bibliographic data supporting the findings of this study are available from the corresponding author upon reasonable request.

## References

[1] Shallis RM Wang R Davidoff A Ma X Zeidan AM. Epidemiology of acute myeloid leukemia: recent progress and enduring challenges. Blood Rev. (2019) 36:70–87. doi: 10.1016/j.blre.2019.04.005, 31101526

[2] Roth JA D'Amico PG Cappelleri JC Donckels EA Yu A Okunev I . Real-world treatment and utilization of venetoclax for incident acute myeloid leukemia in a Medicare population. Future Oncol. (2025) 21:3811–20. doi: 10.1080/14796694.2025.2587568, 41332303 PMC12688266

[3] Docking TR Parker JDK Jädersten M Duns G Chang L Jiang J . A clinical transcriptome approach to patient stratification and therapy selection in acute myeloid leukemia. Nat Commun. (2021) 12:2474. doi: 10.1038/s41467-021-22625-y, 33931648 PMC8087683

[4] Mou T Pawitan Y Stahl M Vesterlund M Deng W Jafari R . The transcriptome-wide landscape of molecular subtype-specific mRNA expression profiles in acute myeloid leukemia. Am J Hematol. (2021) 96:580–8. doi: 10.1002/ajh.26141, 33625756

[5] Kerbs P Vosberg S Krebs S Graf A Blum H Swoboda A . Fusion gene detection by RNA-sequencing complements diagnostics of acute myeloid leukemia and identifies recurring NRIP1-MIR99AHG rearrangements. Haematologica. (2022) 107:100–11. doi: 10.3324/haematol.2021.278436 34134471 PMC8719081

[6] Tyner JW Tognon CE Bottomly D Wilmot B Kurtz SE Savage SL . Functional genomic landscape of acute myeloid leukaemia. Nature. (2018) 562:526–31. doi: 10.1038/s41586-018-0623-z, 30333627 PMC6280667

[7] He F Lin S Gao B Ramesh V Kim AB Kong T . A proteogenomic gene signature defines prognostic subgroups highlighting PI3K/AKT/mTOR signaling pathway as a therapeutic vulnerability in myeloid malignancies. Cell Commun Signal. (2025) 24:61. doi: 10.1186/s12964-025-02568-3 41402890 PMC12853910

[8] Huang J Wang M Zhang Z Chen S. TMEM91::TAL1 fusion gene in a middle-aged female with rapid MDS to secondary AML progression: a case report. Hematology. (2025) 30:2594370. doi: 10.1080/16078454.2025.2594370 41317203

[9] Eldfors S Saad J Ikonen N Malani D Vähä-Koskela M Gjertsen BT . Monosomy 7/del(7q) cause sensitivity to inhibitors of nicotinamide phosphoribosyltransferase in acute myeloid leukemia. Blood Adv. (2024) 8:1621–33. doi: 10.1182/bloodadvances.2023010435, 38197948 PMC10987804

[10] Ng SW Mitchell A Kennedy JA Chen WC McLeod J Ibrahimova N . A 17-gene stemness score for rapid determination of risk in acute leukaemia. Nature. (2016) 540:433–7. doi: 10.1038/nature20598, 27926740

[11] Bottomly D Long N Schultz AR Kurtz SE Tognon CE Johnson K . Integrative analysis of drug response and clinical outcome in acute myeloid leukemia. Cancer Cell. (2022) 40:850–64.e9. doi: 10.1016/j.ccell.2022.07.002, 35868306 PMC9378589

[12] Carraway HE Brunner AM Lai CE Luskin MR Park J Perl AE . Advancing the management of CH, MDS, and AML from the first bridging the gaps in Leukemia, lymphoma, and multiple myeloma conference. Clin Lymphoma Myeloma Leuk. (2025) 25:583–9. doi: 10.1016/j.clml.2025.03.005, 40187939

[13] Ye R Sun M Huang J Sun L Wu Y Zou X . Role and mechanism of the CBX4-HDAC5-CERS6 axis in disrupting sphingomyelin metabolism in acute myeloid leukemia. Am J Cancer Res. (2025) 15:4885–904. doi: 10.62347/VXBP2060, 41395279 PMC12696539

[14] Seo B Kim J Kim S Lee E. Bibliometric analysis of studies about acute myeloid leukemia conducted globally from 1999 to 2018. Blood Res. (2020) 55:1–9. doi: 10.5045/br.2020.55.1.1, 32269969 PMC7106114

[15] Huang W Sun G Wang Q Long Z. The research progress of targeted therapy in acute myeloid leukemia based on bibliometric analysis. Front Oncol. (2022) 12:957370. doi: 10.3389/fonc.2022.957370, 36119476 PMC9481238

[16] Gao Y Wu Z Chen Y Shang G Zeng Y Gao Y. A global bibliometric and visualized analysis of the links between the autophagy and acute myeloid leukemia. Front Pharmacol. (2023) 14:1291195. doi: 10.3389/fphar.2023.1291195, 38322702 PMC10844427

[17] Zhe N Li Q Huang N Li H Chen H Zhu P. Hotspots evolution and frontiers of immunotherapy for the treatment of acute myeloid leukemia: a bibliometric analysis. Hum Vaccin Immunother. (2025) 21:2448888. doi: 10.1080/21645515.2024.2448888, 39819314 PMC12934189

[18] Chen L Wan Y Yang T Zhang Q Zeng Y Zheng S . Bibliometric and visual analysis of single-cell sequencing from 2010 to 2022. Front Genet. (2024) 14:1285599 38274109 10.3389/fgene.2023.1285599 PMC10808606

[19] AlRyalat SAS Malkawi LW Momani SM. Comparing bibliometric analysis using PubMed, Scopus, and web of science databases. J Vis Exp. (2019) 152 10.3791/58494 31710021

[20] Synnestvedt MB Chen C Holmes JH. CiteSpace II: visualization and knowledge discovery in bibliographic databases. AMIA Annu Symp Proc. (2005) 2005:724–8. 16779135 PMC1560567

[21] van Eck NJ Waltman L. Software survey: VOSviewer, a computer program for bibliometric mapping. Scientometrics. (2010) 84:523–38. doi: 10.1007/s11192-009-0146-3, 20585380 PMC2883932

[22] Arruda H Silva ER Lessa M Proença D Jr Bartholo R. VOSviewer and Bibliometrix. J Med Libr Assoc. (2022) 110:392–5. doi: 10.5195/jmla.2022.1434, 36589296 PMC9782747

[23] Ding L Ley TJ Larson DE Miller CA Koboldt DC Welch JS . Clonal evolution in relapsed acute myeloid leukaemia revealed by whole-genome sequencing. Nature. (2012) 481:506–10. doi: 10.1038/nature10738, 22237025 PMC3267864

[24] Jongen-Lavrencic M Grob T Hanekamp D Kavelaars FG Al Hinai A Zeilemaker A . Molecular minimal residual disease in acute myeloid leukemia. N Engl J Med. (2018) 378:1189–99. doi: 10.1056/NEJMoa1716863, 29601269

[25] Ley TJ Miller C Ding L Raphael BJ Mungall AJ Robertson A . Genomic and epigenomic landscapes of adult de novo acute myeloid leukemia. N Engl J Med. (2013) 368:2059–74. doi: 10.1056/NEJMoa1301689, 23634996 PMC3767041

[26] Shlush LI Zandi S Mitchell A Chen WC Brandwein JM Gupta V . Identification of pre-leukaemic haematopoietic stem cells in acute leukaemia. Nature. (2014) 506:328–33. doi: 10.1038/nature13038, 24522528 PMC4991939

[27] Figueroa ME Abdel-Wahab O Lu C Ward PS Patel J Shih A . Leukemic IDH1 and IDH2 mutations result in a hypermethylation phenotype, disrupt TET2 function, and impair hematopoietic differentiation. Cancer Cell. (2010) 18:553–67. doi: 10.1016/j.ccr.2010.11.015, 21130701 PMC4105845

[28] Papaemmanuil E Gerstung M Bullinger L Gaidzik VI Paschka P Roberts ND . Genomic classification and prognosis in acute myeloid Leukemia. N Engl J Med. (2016) 374:2209–21. doi: 10.1056/NEJMoa1516192, 27276561 PMC4979995

[29] Li X Wang CY. From bulk, single-cell to spatial RNA sequencing. Int J Oral Sci. (2021) 13:36. doi: 10.1038/s41368-021-00146-0, 34782601 PMC8593179

[30] van Galen P Hovestadt V Wadsworth MH Ii Hughes TK Griffin GK Battaglia S . Single-cell RNA-seq reveals AML hierarchies relevant to disease progression and immunity. Cell. (2019) 176:1265–81.e24. doi: 10.1016/j.cell.2019.01.031, 30827681 PMC6515904

[31] Wu J Xiao Y Sun J Sun H Chen H Zhu Y . A single-cell survey of cellular hierarchy in acute myeloid leukemia. J Hematol Oncol. (2020) 13:128. doi: 10.1186/s13045-020-00941-y, 32977829 PMC7517826

[32] Baryawno N Przybylski D Kowalczyk MS Kfoury Y Severe N Gustafsson K . A cellular taxonomy of the bone marrow stroma in homeostasis and Leukemia. Cell. (2019) 177:1915–32.e16. doi: 10.1016/j.cell.2019.04.040, 31130381 PMC6570562

[33] Rutella S Vadakekolathu J Mazziotta F Reeder S Yau TO Mukhopadhyay R . Immune dysfunction signatures predict outcomes and define checkpoint blockade-unresponsive microenvironments in acute myeloid leukemia. J Clin Invest. (2022) 132:e159579. doi: 10.1172/JCI159579 36099049 PMC9621145

[34] Pei S Pollyea DA Gustafson A Stevens BM Minhajuddin M Fu R . Monocytic subclones confer resistance to venetoclax-based therapy in patients with acute myeloid leukemia. Cancer Discov. (2020) 10:536–51. doi: 10.1158/2159-8290.cd-19-0710, 31974170 PMC7124979

[35] Heuser M Freeman SD Ossenkoppele GJ Buccisano F Hourigan CS Ngai LL . 2021 update on MRD in acute myeloid leukemia: a consensus document from the European LeukemiaNet MRD working party. Blood. (2021) 138:2753–67. doi: 10.1182/blood.2021013626, 34724563 PMC8718623

[36] Lambo S Trinh DL Ries RE Jin D Setiadi A Ng M . A longitudinal single-cell atlas of treatment response in pediatric AML. Cancer Cell. (2023) 41:2117–35.e12. doi: 10.1016/j.ccell.2023.10.008, 37977148

[37] Li K Du Y Cai Y Liu W Lv Y Huang B . Single-cell analysis reveals the chemotherapy-induced cellular reprogramming and novel therapeutic targets in relapsed/refractory acute myeloid leukemia. Leukemia. (2023) 37:308–25. doi: 10.1038/s41375-022-01789-6, 36543880 PMC9898038

[38] Goossens N Nakagawa S Sun X Hoshida Y. Cancer biomarker discovery and validation. Transl Cancer Res. (2015) 4:256–69. doi: 10.3978/j.issn.2218-676X.2015.06.04 26213686 PMC4511498

[39] Conesa A Madrigal P Tarazona S Gomez-Cabrero D Cervera A McPherson A . A survey of best practices for RNA-seq data analysis. Genome Biol. (2016) 17:13. doi: 10.1186/s13059-016-0881-8, 26813401 PMC4728800

[40] Fisher NC Byrne RM Leslie H Wood C Legrini A Cameron AJ . Biological misinterpretation of transcriptional signatures in tumor samples can unknowingly undermine mechanistic understanding and faithful alignment with preclinical data. Clin Cancer Res. (2022) 28:4056–69. doi: 10.1158/1078-0432.CCR-22-1102, 35792866 PMC9475248

[41] Mereu E Lafzi A Moutinho C Ziegenhain C McCarthy DJ Álvarez-Varela A . Benchmarking single-cell RNA-sequencing protocols for cell atlas projects. Nat Biotechnol. (2020) 38:747–55. doi: 10.1038/s41587-020-0469-4 32518403

[42] Kim J Schanzer N Singh RS Zaman MI Garcia-Medina JS Proszynski J . DOGMA-seq and multimodal, single-cell analysis in acute myeloid leukemia. Int Rev Cell Mol Biol. (2025) 390:67–108. doi: 10.1016/bs.ircmb.2024.08.001, 39864897 PMC12368869

[43] Mimitou EP Lareau CA Chen KY Zorzetto-Fernandes AL Hao Y Takeshima Y . Scalable, multimodal profiling of chromatin accessibility, gene expression and protein levels in single cells. Nat Biotechnol. (2021) 39:1246–58. doi: 10.1038/s41587-021-00927-2, 34083792 PMC8763625

[44] Cai Z Poulos RC Liu J Zhong Q. Machine learning for multi-omics data integration in cancer. iScience. (2022) 25:103798. doi: 10.1016/j.isci.2022.103798, 35169688 PMC8829812

[45] Collins GS Moons KGM Dhiman P Riley RD Beam AL Van Calster B . TRIPOD+AI statement: updated guidance for reporting clinical prediction models that use regression or machine learning methods. BMJ. (2024) 385:e078378. doi: 10.1136/bmj-2023-078378, 38626948 PMC11019967

[46] Moons KGM Damen JAA Kaul T Hooft L Andaur Navarro C Dhiman P . PROBAST+AI: an updated quality, risk of bias, and applicability assessment tool for prediction models using regression or artificial intelligence methods. BMJ. (2025) 388:e082505. doi: 10.1136/bmj-2024-082505, 40127903 PMC11931409

[47] Rao A Barkley D França GS Yanai I. Exploring tissue architecture using spatial transcriptomics. Nature. (2021) 596:211–20. doi: 10.1038/s41586-021-03634-9, 34381231 PMC8475179

[48] Moses L Pachter L. Museum of spatial transcriptomics. Nat Methods. (2022) 19:534–46. doi: 10.1038/s41592-022-01409-2, 35273392

[49] Heumos L Schaar AC Lance C Litinetskaya A Drost F Zappia L . Best practices for single-cell analysis across modalities. Nat Rev Genet. (2023) 24:550–72. doi: 10.1038/s41576-023-00586-w, 37002403 PMC10066026

[50] Tran HTN Ang KS Chevrier M Zhang X Lee NYS Goh M . A benchmark of batch-effect correction methods for single-cell RNA sequencing data. Genome Biol. (2020) 21:12. doi: 10.1186/s13059-019-1850-9, 31948481 PMC6964114

[51] Teutsch SM Bradley LA Palomaki GE Haddow JE Piper M Calonge N . The evaluation of genomic applications in practice and prevention (EGAPP) initiative: methods of the EGAPP working group. Genet Med. (2009) 11:3–14. doi: 10.1097/GIM.0b013e318184137c, 18813139 PMC2743609

