# Supplementary material for: From transcriptomic profiling to precision oncology: a bibliometric analysis of RNA sequencing in acute myeloid leukemia
Source: Front Med (Lausanne). 2026 Aug 27;13:1877223. doi: 10.3389/fmed.2026.1877223 (PMC13557956; doi:10.3389/fmed.2026.1877223)
Supplement: Supplementary file 1 [file Table_1.docx]

**RNA sequencing in acute myeloid leukemia: A bibliometric analysis (2007–2025)**

**Running title: RNA-Seq in AML**

**Supplemental Tables**

**Supplementary Table S1. Bibliometric Overview of RNA Sequencing in Acute Myeloid Leukemia (2007–2025)**

**Supplementary Table S2. Most Relevant Countries by Corresponding Author Contributions (2007–2025)**

**Supplementary Table S3: Top 5 Authors on RNA Sequencing in Acute Myeloid Leukemia (2007–2025)**

**Supplementary Table S4: Top 10 Influential Journals on RNA Sequencing in Acute Myeloid Leukemia (2007–2025)**

**Supplementary Table S5: Top 10 Most Cited Publications Based on Bibliometrix Analysis (2007–2025)**

**Supplementary Table S1**. **Bibliometric Overview of RNA Sequencing in Acute Myeloid Leukemia (2007–2025)**

| **Description** | **Results** |
| --- | --- |
| MAIN INFORMATION ABOUT DATA |  |
| Timespan | 2007:2025 |
| Sources (Journals, Books, etc) | 694 |
| Documents | 3460 |
| Annual Growth Rate % | 39.78 |
| Document Average Age | 3.66 |
| Average citations per doc | 24.42 |
| References | 145422 |
| DOCUMENT CONTENTS |  |
| Keywords Plus (ID) | 17848 |
| Author's Keywords (DE) | 5458 |
| AUTHORS |  |
| Authors | 22339 |
| Authors of single-authored docs | 41 |
| AUTHORS COLLABORATION |  |
| Single-authored docs | 42 |
| Co-Authors per Doc | 11.4 |
| International co-authorships % | 13.84 |
| DOCUMENT TYPES |  |
| article | 3360 |
| review | 100 |

**Supplementary Table S2. Most Relevant Countries by Corresponding Author Contributions (2007–2025)**

| **Rank** | **Country** | **Articles** | **Articles %** | **SCP** | **MCP** | **MCP %** |
| --- | --- | --- | --- | --- | --- | --- |
| 1 | CHINA | 547 | 15.8 | 477 | 70 | 12.8 |
| 2 | USA | 358 | 10.3 | 229 | 129 | 36 |
| 3 | GERMANY | 90 | 2.6 | 48 | 42 | 46.7 |
| 4 | UNITED KINGDOM | 59 | 1.7 | 14 | 45 | 76.3 |
| 5 | CANADA | 54 | 1.6 | 31 | 23 | 42.6 |
| 6 | ITALY | 52 | 1.5 | 26 | 26 | 50 |
| 7 | KOREA | 52 | 1.5 | 47 | 5 | 9.6 |
| 8 | FRANCE | 37 | 1.1 | 27 | 10 | 27 |
| 9 | JAPAN | 33 | 1 | 25 | 8 | 24.2 |
| 10 | NETHERLANDS | 28 | 0.8 | 12 | 16 | 57.1 |
| 11 | SWEDEN | 28 | 0.8 | 17 | 11 | 39.3 |
| 12 | NORWAY | 25 | 0.7 | 22 | 3 | 12 |
| 13 | AUSTRALIA | 22 | 0.6 | 7 | 15 | 68.2 |
| 14 | INDIA | 19 | 0.5 | 14 | 5 | 26.3 |
| 15 | SPAIN | 17 | 0.5 | 10 | 7 | 41.2 |
| 16 | FINLAND | 10 | 0.3 | 4 | 6 | 60 |
| 17 | IRAN | 9 | 0.3 | 6 | 3 | 33.3 |
| 18 | AUSTRIA | 8 | 0.2 | 2 | 6 | 75 |
| 19 | BELGIUM | 8 | 0.2 | 3 | 5 | 62.5 |
| 20 | DENMARK | 8 | 0.2 | 3 | 5 | 62.5 |
| 21 | POLAND | 8 | 0.2 | 7 | 1 | 12.5 |
| 22 | CZECH REPUBLIC | 7 | 0.2 | 3 | 4 | 57.1 |
| 23 | RUSSIA | 7 | 0.2 | 6 | 1 | 14.3 |
| 24 | BRAZIL | 6 | 0.2 | 3 | 3 | 50 |
| 25 | SINGAPORE | 6 | 0.2 | 4 | 2 | 33.3 |

**Abbreviation:** SCP: Single Country Publications; MCP: Multiple Country Publications;

**Supplementary Table S3: Top 5 Authors on RNA Sequencing in Acute Myeloid Leukemia (2007–2025)**

| **Rank** | **Author** | **h_index** | **g_index** | **m_index** | **TC** | **NP** | **PY_start** | **Articles** | **Articles Fractionalized** |
| --- | --- | --- | --- | --- | --- | --- | --- | --- | --- |
| 1 | LIU Y | 22 | 50 | 1.833 | 2576 | 94 | 2014 | 94 | 8.43 |
| 2 | WANG J | 22 | 39 | 1.833 | 1755 | 97 | 2014 | 97 | 8.54 |
| 3 | BULLINGER L | 18 | 27 | 1.5 | 1399 | 27 | 2014 | 27 | 1.96 |
| 4 | WANG Y | 18 | 36 | 1.636 | 1545 | 99 | 2015 | 99 | 10.24 |
| 5 | ZHANG J | 18 | 44 | 1 | 1999 | 59 | 2008 | 59 | 5.13 |

**Abbreviation:**TC: Total Citations; NP: Number of Publications; PY_start: Year of First Publication

**Supplementary Table S4: Top 10 Influential Journals on RNA Sequencing in Acute Myeloid Leukemia (2007–2025)**

| **Rank** | **Source** | **h_index** | **g_index** | **m_index** | **TC** | **NP** | **PY_start** | **IF** | **JCR** |
| --- | --- | --- | --- | --- | --- | --- | --- | --- | --- |
| 1 | BLOOD | 45 | 75 | 2.813 | 6000 | 111 | 2010 | 23.1 | Q1 |
| 2 | LEUKEMIA | 38 | 56 | 2.533 | 3839 | 145 | 2011 | 13.4 | Q1 |
| 3 | NATURE COMMUNICATIONS | 36 | 62 | 3 | 3957 | 82 | 2014 | 15.7 | Q1 |
| 4 | HAEMATOLOGICA | 25 | 41 | 2.083 | 1949 | 73 | 2014 | 7.9 | Q1 |
| 5 | BLOOD ADVANCES | 23 | 33 | 2.875 | 1398 | 80 | 2018 | 7.1 | Q1 |
| 6 | CANCER DISCOVERY | 23 | 30 | 2.3 | 2309 | 30 | 2016 | 33.3 | Q1 |
| 7 | CANCER CELL | 20 | 23 | 1.818 | 2550 | 23 | 2015 | 44.5 | Q1 |
| 8 | CANCER RESEARCH | 18 | 31 | 1.5 | 962 | 34 | 2014 | 16.6 | Q1 |
| 9 | FRONTIERS IN ONCOLOGY | 18 | 27 | 1.636 | 1058 | 97 | 2015 | 3.3 | Q2 |
| 10 | PLOS ONE | 18 | 31 | 1.125 | 1002 | 44 | 2010 | 2.6 | Q2 |

**Abbreviation:** IF: Impact Factor; JCR: Journal Citation Reports

**Supplementary Table S5: Top 10 Most Cited Publications Based on Bibliometrix Analysis (2007–2025)**

| **Rank** | **First Author** | **Year** | **Journal** | **Paper** | **DOI** | **Total Citations** | **TC per Year** | **Normalized TC** |
| --- | --- | --- | --- | --- | --- | --- | --- | --- |
| 1 | DING L | 2012 | NATURE | Clonal evolution in relapsed acute myeloid leukaemia revealed by whole-genome sequencing | 10.1038/nature10738 | 1698 | 121.29 | 7.92 |
| 2 | TYNER JW | 2018 | NATURE | Functional genomic landscape of acute myeloid leukaemia | 10.1038/s41586-018-0623-z | 930 | 116.25 | 12.67 |
| 3 | BARBIERI I | 2020 | NAT REV CANCER | Role of RNA modifications in cancer | 10.1038/s41568-020-0253-2 | 914 | 152.33 | 22.74 |
| 4 | VAN GALEN P | 2019 | CELL | Single-Cell RNA-Seq Reveals AML Hierarchies Relevant to Disease Progression and Immunity | 10.1016/j.cell.2019.01.031 | 722 | 103.14 | 16.29 |
| 5 | JONGEN-LAVRENCIC M | 2018 | NEW ENGL J MED | Molecular Minimal Residual Disease in Acute Myeloid Leukemia | 10.1056/NEJMoa1716863 | 653 | 81.63 | 8.89 |
| 6 | MA XT | 2018 | NATURE | Pan-cancer genome and transcriptome analyses of 1,699 paediatric leukaemias and solid tumours | 10.1038/nature25795 | 621 | 77.63 | 8.46 |
| 7 | BARYAWNO N | 2019 | CELL | A Cellular Taxonomy of the Bone Marrow Stroma in Homeostasis and Leukemia | 10.1016/j.cell.2019.04.040 | 612 | 87.43 | 13.81 |
| 8 | WU S | 2020 | MOL CANCER | Natural killer cells in cancer biology and therapy | 10.1186/s12943-020-01238-x | 585 | 97.50 | 14.55 |
| 9 | ARDUI S | 2018 | NUCLEIC ACIDS RES | Single molecule real-time (SMRT) sequencing comes of age: applications and utilities for medical diagnostics | 10.1093/nar/gky066 | 566 | 70.75 | 7.71 |
| 10 | LIN MJ | 2022 | NATURE CANCER | Cancer vaccines: the next immunotherapy frontier | 10.1038/s43018-022-00418-6 | 559 | 139.75 | 31.20 |

**Abbreviation:** DOI: Digital Object Identifier
